# Recent advances of sterile inflammation and inter-organ cross-talk in alcoholic liver disease

**DOI:** 10.1038/s12276-020-0438-5

**Published:** 2020-05-26

**Authors:** Young-Ri Shim, Won-Il Jeong

**Affiliations:** Laboratory of Liver Research, Graduate School of Medical Science and Engineering, Korea Advanced Institute of Science and Technology, Daejeon, Korea

**Keywords:** Cell biology, Alcoholic liver disease

## Abstract

Alcoholic liver disease (ALD) is one of the fastest-growing concerns worldwide. In addition to bacterial endotoxins in the portal circulation, recent lines of evidence have suggested that sterile inflammation caused by a wide range of stimuli induces alcoholic liver injury, in which damage-associated molecular patterns (DAMPs) play critical roles in inducing de novo lipogenesis and inflammation through the activation of cellular pattern recognition receptors such as Toll-like receptors in non-parenchymal cells. Interestingly, alcohol-mediated metabolic, neurological, and immune stresses stimulate the generation of DAMPs that are released not only in the liver, but also in other organs, such as adipose tissue, intestine, and bone marrow. Thus, diverse DAMPs, including retinoic acids, proteins, lipids, microRNAs, mitochondrial DNA, and mitochondrial double-stranded RNA, contribute to a broad spectrum of ALD through the production of multiple pro-inflammatory cytokines, chemokines, and ligands in non-parenchymal cells, such as Kupffer cells, hepatic stellate cells, and various immune cells. Therefore, this review summarizes recent studies on the identification and understanding of DAMPs, their receptors, and cross-talk between the liver and other organs, and highlights successful therapeutic targets and potential strategies in drug development that can be used to combat ALD.

## Alcoholic liver disease: cross-talk with other organs

Therapies preventing the distribution of damage-associated molecules from other organs to the liver could help tackle alcoholic liver disease (ALD). Over time, metabolites from excessive alcohol consumption induce oxidative stress, damaging liver cells. Injured hepatocytes release molecules known as damage-associated molecular patterns (DAMPs), including proteins, RNA, and metabolites, which disperse within the liver and other organs and trigger chronic inflammation. Won-Il Jeong and Young-Ri Shim at the Korea Advanced Institute of Science and Technology in Daejeon reviewed understanding of DAMPs to identify possible therapeutic targets for ALD. DAMPs are carried between cells by extracellular vesicles, particles released during cellular communication. Alcohol-induced DAMPs in other organs, such as the gut, can deliver to the liver, and it influences ALD progression. The more-detailed cross-talk between the liver and other organs in ALD requires further investigation.

## Introduction

Chronic alcohol consumption is the third highest health risk in the world. The World Health Organization has reported that the abuse of alcohol kills up to three million people per year globally; it accounts for ~5% of the global disease burden in 2018^1,2^. Alcoholic liver disease (ALD) progresses from a mild form of alcoholic fatty liver to severe forms, such as alcoholic steatohepatitis, alcoholic hepatitis, alcoholic fibrosis/cirrhosis, and hepatocellular carcinoma (HCC)^2,3^.

In general, three inflammatory pathways primarily trigger ALD, wherein a change in intestinal microbiome composition increases the amount of pathogen-associated molecular patterns (PAMPs) that further mediate activation of Kupffer cells through pattern recognition receptors (PRRs). Moreover, damage to hepatocytes by alcohol metabolites generates reactive oxygen species (ROS), lipid-originated metabolites (retinoic acid and endocannabinoids), and damage-associated molecular patterns (DAMPs) to stimulate inflammatory signals through Toll-like receptors (TLRs), nuclear/neuronal receptors, and the inflammasome^2,4^. Furthermore, inter-organ cross-talk contributes to ALD by delivering DAMPs or migrating inflammatory cells to the liver. Through these processes, hepatocytes and non-parenchymal cells produce pro-inflammatory cytokines and chemokines to recruit additional immune cells, such as neutrophils and macrophages. In addition, diverse types of hepatic lymphocytes that are activated by PAMPs, DAMPs, and cytokines promote liver injury by producing interferon (IFN)-γ, interleukin (IL)-22, IL-17, etc^5^. Recent emerging data on sterile inflammation may bring to light potential therapeutic targets for ALD. Thus, this review describes the current state of understanding concerning the pathophysiological mechanisms of DAMP- and PAMP-mediated inflammation and organ cross-talk in ALD. In addition, we briefly summarize lists of DAMPs and PAMPs in ALD (Table 1).

**Table 1:** DAMPs and PAMPs in ALD.

Origin	Ligands	Receptors	Functions	Cells	Refs.
DAMPs	Glutamate	mGluR5	2-AG production	HSC	^24^
	HMGB1	TLR4, RAGE	Post-translational modification	Macrophage/Kupffer cell	^7^
	ATP	P2X7	Inflammasome activation	Macrophage/Kupffer cell	^9^
	MicroRNA (miRNA)	‒	M1 polarization	Macrophage/Kupffer cell	^14^
	Mitochondrial dsRNA	TLR3	IL-1β	Macrophage	^16^
	Mitochondrial DNA	TLR9, NLRP3	IL-1β, IL-17A	Kupffer cell/neutrophil/tumor cell	^8,20^
	Nuclear (apoptotic) DNA	TLR9	TGF-β, collagen	HSC	^19^
	EV ligands (miRNA, CD40L, HSP90)	CD40	TNF-α, IL-1β, M1 polarization	Macrophage/Kupffer cell	^13,21,22^
	Metabolites (RAs)	RARs, RXRs	RAE, IFN-γ	HSCs	^27,29–31^
	Lipids (FFAs, TG)	CD36	Lipotoxicity	Hepatocyte	^42,57^
PAMPs	LPS	TLR4	TNF-α, IL-1β	Kupffer cell	^35,36^
	LTA	TLR2	TNF-α	Kupffer cell	^36^
	CpG DNA	TLR9	TNF-α	Kupffer cell	^21^
	Flagellin	TLR5	TNF-α, IL-22	Kupffer cell, Immune cell at the ileum	^36,37^
	β-glucan	CLEC7A	IL-1β	Kupffer cell	^87^

*mGluR5* metabotropic glutamate receptor 5, *2-AG* 2-Arachidonoylglycerol, *HSC* hepatic stellate cell, *HMGB1* high mobility group box-1, *TLR* toll-like receptor, *ATP* adenosine triphosphate, *IL* interleukin, *NLRP3* NLR family pyrin domain containing 3, *TGF* transforming growth factor, *EV* extracellular vesicle, *CD40L* CD40 ligand, *HSP90* heat shock protein 90, *TNF* tumor necrosis factor, *RA* retinoic acid, *RAR* retinoic acid receptors, *RXRs* retinoid X receptor, *RAE* retinoic acid early inducible, *IFN* interferon, *FFA* free fatty acid, *TG* triglyceride, *LPS* lipopolysaccharide, *LTA* lipoteichoic acid, *CLEC7A* C-type lectin domain containing 7A.

## Damps in ALD

### Alcohol metabolism and DAMPs

In the early stage of alcohol consumption, alcohol is metabolized to acetaldehyde by alcohol dehydrogenase, which is further converted to acetate by acetaldehyde dehydrogenase^6^; however, chronic exposure results in alcohol metabolism occurring mainly through cytochrome P450 2E1 (CYP2E1)^7^. In the late stage, alcohol metabolism-induced oxidative stresses cause damage to hepatocytes, in which DAMPs, including high mobility group box-1 (HMGB1), mitochondrial DNA (mtDNA), mitochondrial double-stranded RNA (mtdsRNA), microRNAs (miRNAs), ATP, and several metabolites are generated and released from the injured hepatocytes, leading to sterile inflammation in ALD^8–10^. In addition to recognizing TLRs, DAMPs amplify liver injury by stimulation of the inflammasome, which activates caspase-1 and secretes the pro-inflammatory cytokines, such as IL-1β and IL-18, which play important roles in ALD^5,11,12^.

### Extracellular vesicles (EVs), miRNA, mtdsRNA, and mtDNA in ALD

EVs, such as exosomes, microvesicles, and apoptotic bodies, play important roles in cell-to-cell communication by delivering hepatic DAMPs to target cells^13^. Recent studies in mice and patients with alcoholic steatohepatitis (ASH) have demonstrated that many miRNAs are not only generated within the cells but can also be transferred into other target cells via EVs^14^. For instance, miR-27a from alcohol-exposed monocytes can program naive monocytes to polarize into M2 macrophages^15^. In addition, mtdsRNA can be generated by the inhibition of restricting enzymes, such as mitochondrial RNA helicase SUV3 and polynucleotide phosphorylase (PNPase)^16^. We recently found that accumulation of mtdsRNA is induced in ethanol-exposed hepatocytes through downregulated expression of PNPase, and exosomal delivery of mtdsRNA to Kupffer cells leads to an augmentation of IL-17 production in hepatic γδ T cells and the severity of acute ALD in a TLR3-dependent manner^17^. In RNA-sequencing analysis, most mitochondrial mRNAs were enhanced in ethanol-exposed exosomes, whereas mitochondrial DNAs were mainly enriched in microvesicles after treating hepatocytes with ethanol in vitro^17^. Another study strongly suggested that hepatocyte mtdsRNA could act as self-ligands to TLR3, which could aggravate not only ALD but also other liver diseases^17,18^. In contrast, poly I:C-mediated multiple activation of TLR3 in Kupffer cells and hepatic stellate cells (HSCs) attenuates hepatic steatosis and inflammation via IL-10 production^19^. Thus, the roles of TLR3 should be investigated carefully in further studies. Similarly, previous studies have reported that nuclear DNA and mtDNA of damaged hepatocytes contribute to ALD. For example, EVs deliver hepatocyte-derived apoptotic DNA and mtDNA to HSCs, neutrophils or tumor cells, consequently accelerating liver fibrosis, alcoholic hepatitis, and HCC, respectively, through TLR9^9,20,21^. In addition to nucleic acids, CD40 ligands and heat shock protein 90 present in EVs stimulate the production of TNF-α and IL-1β in macrophages in mice and patients with alcoholic hepatitis, in vitro and in vivo^22,23^. Therefore, EVs could be therapeutic targets for ALD.

### Glutamate and endocannabinoids in ALD

During the last decade, studies have demonstrated that the bidirectional cross-talk between hepatocytes and HSCs contributes to alcoholic steatosis, in which a line of neurological responses between hepatocytes and HSCs suggests the presence of a metabolic synapse^24,25^. Alcoholic steatosis is mediated by activation of the endocannabinoid-induced CB_1_ receptor (CB_1_R) in hepatocytes, in which the metabotropic glutamate receptor 5 (mGluR5) in HSCs interacts with hepatocyte-derived glutamate and generates the endocannabinoid, 2-arachidonoylglycerol (2-AG), after alcohol exposure^25^. Chronic alcohol exposure induces CYP2E1-mediated oxidative stress, which suppresses the methionine cycle and transsulfuration system, and decreases cysteine concentrations hepatocytes, thereby lowering the levels of the antioxidant molecule glutathione (GSH)^26^. Moreover, to compensate for the GSH shortage, hepatocytes take up extracellular cystine by exchanging it with glutamate through the xCT transporter, whereas HSCs generate 2-AG through mGluR5-mediated diacylglycerol lipase-β activation^25^. The produced 2-AG, in turn, stimulates hepatic CB_1_R to induce de novo lipogenesis by upregulating sterol regulatory element-binding protein 1c (SREBP1c) and downregulating AMP-activated protein kinase in hepatocytes, leading to fat accumulation. Furthermore, we found that chronic alcohol consumption increases glutamate generation from glutamic-γ-semialdehyde through upregulation of ALDH4A1 expression in perivenous hepatocytes^25^, suggesting that the glutamate released from damaged hepatocytes might be a type of DAMP. Therefore, a functional metabolic synapse between hepatocytes and HSCs may be a critical therapeutic target for ALD.

### Retinoic acids in ALD

The liver is a representative organ for storage and metabolism of retinol (vitamin A)^27^. In particular, quiescent HSCs store ~80% of total liver retinols as retinyl palmitate in fat droplets, whereas activated HSCs lose their retinols by releasing or metabolizing retinols into retinoids, including retinoic acids (RAs), in response to liver injury^2,27^. In HSCs, a class III ADH3 enzyme plays a critical role in retinol metabolism, and the metabolized RAs bind to retinoic acid receptors (RAR-α/β/γ) and retinoic X receptors (RXR-α/β/γ) and regulate gene expression^2,28^. Although the exact underlying mechanism by which HSCs lose or metabolize retinols in ALD is still unclear, HSCs do not induce liver fibrosis after chronic ethanol consumption in mice^2^. It is probable that activated HSCs express retinoic acid early inducible gene 1 (RAE1), which is a specific ligand for natural killer group 2 member D (NKG2D) in natural killer (NK) cells, and then hepatic NK cells specifically kill the activated HSCs by producing IFN-γ in response to the RAE1-NKG2D interaction at an early stage of ALD^2,29^. In contrast, in an advanced stage of liver disease, activated HSCs show resistance to NK cell cytotoxicity via 9-cis forms of retinoid-mediated production of transforming growth factor (TGF)-β1 and the expression of suppressor of cytokine signaling protein 1 (SOCS1)^29–31^. In addition, a study reported that retinol metabolism in HSCs inhibits the recruitment of regulatory T cells (Tregs), whereas blocking retinol metabolism mitigates Concanavalin A-induced hepatitis through increased migration of Tregs in mice^32^. As mentioned above, liver diseases including ALD stimulate retinol metabolism of HSCs, and retinoids trigger transcription of diverse genes through their nuclear receptors, inducing beneficial or detrimental functions related to liver diseases. However, to implicate retinols as DAMPs in ALD, the exact molecular signaling pathways of retinol metabolism in HSCs and functions in the cells should be clearly investigated, and then these molecules might be therapeutic targets for ALD in the future.

### PAMPS in ALD

The liver–gut axis is a critical pathway for ALD because it is a central player in the response to gut bacteria-originated PAMPs, as well as nutrients received through the portal vein. In this regard, ALD does not represent a true sterile inflammatory liver disease. Thus, we briefly address the role of PAMPs in ALD here. Alcohol consumption affects multiple defense barriers, including chemical, physical, and immune factors in the gut^4^. Impairment of this barrier owing to acute, binge, or chronic alcohol intake increases blood levels of bacterial components and their products (e.g., PAMPs) in animal models and humans^33,34^. Among bacterial PAMPs, lipopolysaccharide (LPS) and bacterial DNA interact with TLR4 and TLR9, respectively, and produce pro-inflammatory cytokines such as tumor necrosis factor (TNF)-α and IL-1β through NF-κB in innate immune cells, including Kupffer cells. Similarly, these PAMPs and inflammatory mediators, such as TNF-α, IL-6, and CCL2, are increased in the sera of humans after alcohol exposure and exposure to LPS^33,34^. In addition, alcohol intake increases serum levels of lipoteichoic acid (LTA) and flagellin, and it sensitizes LTA and flagellin to TLR2 and TLR5, respectively, leading to enhanced TNF-α production and liver injury in a mouse model of ALD^35^. More intriguingly, non-alcoholic fatty liver disease (NAFLD) is also triggered by sterile inflammatory responses, and a recent interesting study in humans suggests that the presence of *Klebsiella pneumoniae* in the gut microbiome might be one of the causes of NAFLD because of its ability to produce alcohol, thereby increasing the alcohol concentration in the blood^36^.

## Inter-organ cross-talk in ALD

**Fig. 1:**
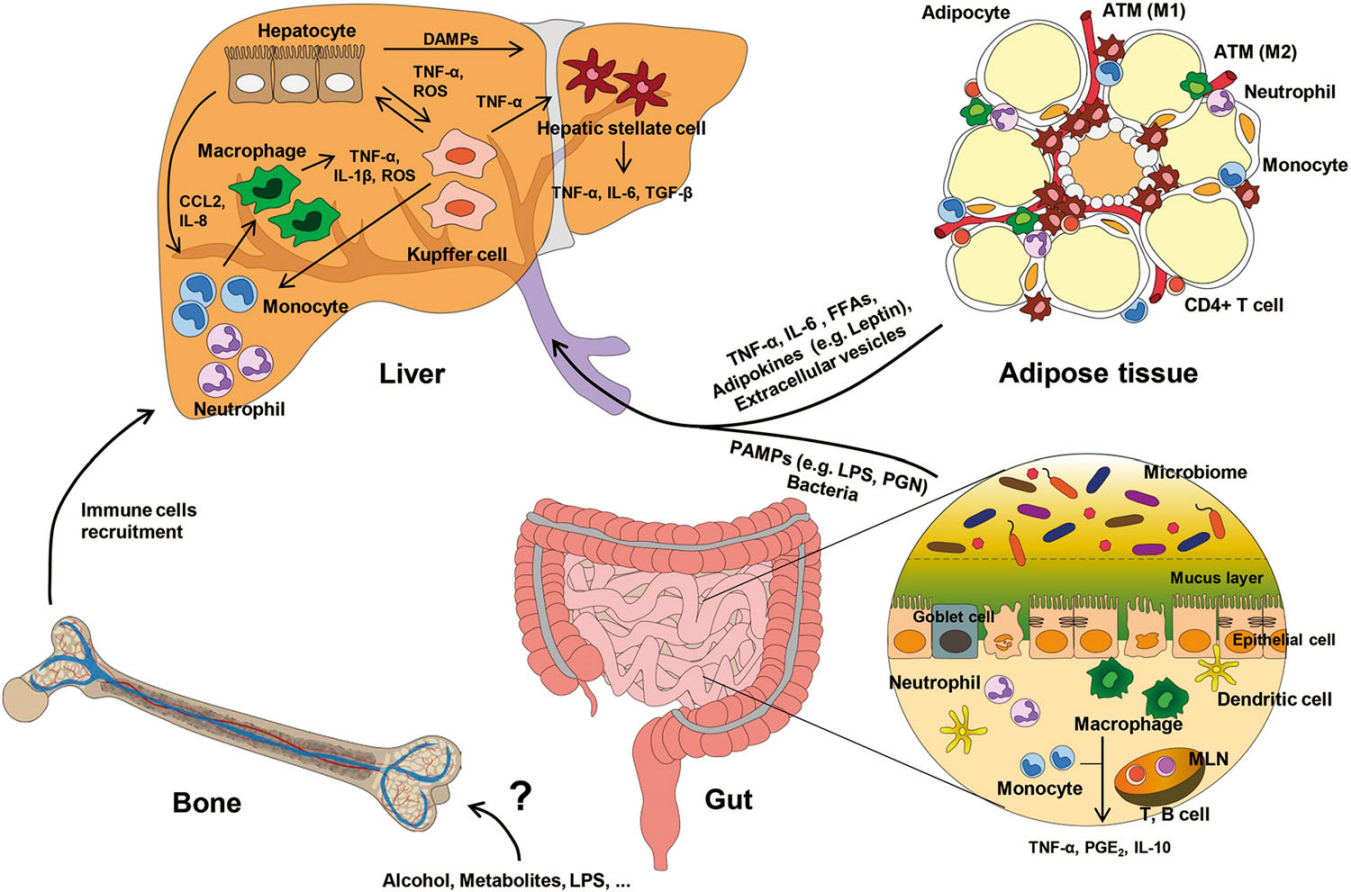
Inter-organ cross-talk in alcoholic liver disease. Alcohol consumption induces lipolysis in adipocytes and inflammatory responses in adipose immune cells, including macrophages, which in turn lead to the release of free fatty acids (FFAs), adipokines (e.g., leptin), and cytokines (e.g., TNF-α and IL-6) into the portal circulation. In addition, alcohol intake alters the gut microbiome composition and increases the permeability of intestinal bacteria and their metabolites through broken barriers of epithelial cells in the gut, thus leading to translocation of bacteria and inflammatory cells in the liver. Therefore, metabolic and immunogenic factors, including DAMPs and PAMPs, from adipose tissue and the gut enter the liver, affecting hepatocytes and non-parenchymal cells in the liver to recruit pro-inflammatory immune cells from the bone marrow. Taken together, inter-organ cross-talk between the liver and other organs plays a critical role in the pathogenesis of ALD.

Several lines of evidence have reported cross-talk between the liver and other organs, such as adipose tissue, the gut, and bone marrow (BM, Fig. 1). For example, metabolic and genetic changes in adipocytes and enterocytes affect steatosis and inflammation of the liver. In addition, altered gene expression in hepatocytes and non-parenchymal cells in the liver influences adipogenesis, lipolysis, and inflammation of adipose tissue, the gut, or BM.

## The adipose tissue-liver axis in ALD

### Metabolic effects of alcohol in adipose tissue

Anatomically, adipose tissue consists of visceral adipose tissue (VAT) and subcutaneous adipose tissue^37^. VAT is mainly present within the abdominal cavity, and visceral fat venous blood is drained directly into the liver through the portal vein. Thus, abnormal metabolic pathways and inflammation in VAT are implicated in the pathogenesis of metabolic syndromes, including obesity, diabetes, atherosclerosis, and NAFLD^38^. White adipocytes, which account for most adipocytes, function to store energy as triglycerides in large lipid droplets and release adipokines and cytokines for metabolic and endocrine activities. In contrast, brown adipocytes contribute to thermogenesis through the high expression of uncoupling protein 1 in mitochondria^39^. Recent studies have suggested that chronic alcohol consumption is inversely correlated with fat accumulation in adipose tissue. In mice and rats, chronic alcohol exposure stimulates adipose triglyceride lipase (ATGL)-mediated lipolysis in adipose tissue, leading to the release of free fatty acids (FFAs) and a decrease in the epididymal adipose tissue mass and adipocyte size^40^. Moreover, binge drinking or chronic alcohol consumption impairs insulin sensitivity, thus resulting in increased lipolysis and decreased lipogenesis^41^. The expression of lipogenic enzymes, peroxisome proliferator-activated receptor-γ, and CCAAT enhancer-binding protein α, is also downregulated in white adipose tissue after chronic alcohol intake^40^. Interestingly, alcohol can be metabolized in adipose tissue in humans and rodents owing to the expression of CYP2E1 and ALDH^42^. In adipose tissue, increased CYP2E1 expression owing to chronic alcohol intake decreases the GSH/GSSG ratio, induces oxidative stress through ROS production, and inhibits adiponectin secretion^42^. Leptin (an energy expenditure hormone) is also known to be related to alcohol intake, but its expression depends on the amount and duration of alcohol consumption in ALD patients and a rodent model^43,44^.

### Inflammation in adipose tissue

Chronic alcohol consumption stimulates the secretion of several cytokines and chemokines, thereby inducing inflammation in adipose tissue. In alcoholic patients, the expression of pro-inflammatory cytokines (e.g., IL-6 and TNF-α) and chemokines (e.g., CCL2), is upregulated in adipose tissue^45,46^. IL-6 expression increases in all stages of ALD, whereas increased expression of TNF-α is only observed in patients with alcoholic steatosis and alcoholic hepatitis^37^. Rodent models also show increased expression of inflammatory mediators, such as TNF-α, IL-6, CCL2, and IFN-γ, in adipose tissue^46^. In adipose tissue inflammation, the number of macrophages is increased (up to 4–50%), in which adipose tissue macrophages (ATMs) are divided into M1 and M2 types^47^. M1 macrophages exist as crown-like structures around dying adipocytes and classically express inflammatory cytokines, such as TNF-α, IL-6, and inducible nitric oxide synthase (iNOS), whereas the M2 type is an alternatively activated cell type that produces anti-inflammatory cytokines, such as IL-10, IL-4, and arginase 1. In a normal state, most ATMs exist as the M2 type; however, in a state of alcohol consumption, these cells shift to the M1 type, expressing CD11c and producing inflammatory cytokines or causing infiltration of M1 macrophages in a CCL2-dependent manner^46^. In addition, binge alcohol intake increases the infiltration of neutrophils with neutrophil-attracting chemokines, thereby inducing tissue damage in mice^48^. In contrast, IL-10 levels in adipose tissue are increased in acute alcoholic hepatitis, suggesting that some cells possess anti-inflammatory functions that regulate the immune system^49^.

### Adipocyte death

Adipocyte death is induced by chronic inflammation and oxidative stress. The ATMs surround dead adipocytes in crown-like structures, and they produce pro-inflammatory cytokines such as TNF-α and IL-6 to facilitate phagocytosis or scavenging of adipocytes^50^. Moreover, it has been reported that chronic ethanol consumption increases Bid-mediated apoptosis in adipocytes, whereas CYP2E1 deficiency results in decreased expression of TNF-α, IL-6, and CCL2 and reduced adipocyte apoptosis in mice^46^, suggesting that adipose tissue inflammation is dependent on CYP2E1-mediated alcohol metabolism. Thus, CYP2E1 might be a good therapeutic target in adipose tissue.

### Cross-talk between the liver and adipose tissue

Increased amounts of circulating FFAs caused by lipolysis in adipocytes contribute to lysosomal destabilization in hepatocytes, leading to TNF-α production and hepatic de novo lipogenesis through transcription of SREBP1c^51^. Moreover, among adipokines, adiponectin in humans and rodents contributes to not only the oxidization of fatty acids in hepatocytes but also the reduction in TNF-α and IL-10 production in Kupffer cells, thereby alleviating liver steatosis and inflammation^52^. The leptin from adipose tissue induces hepatic inflammation and fibrogenic responses by activating HSCs and Kupffer cells, and Kupffer cells increase TNF-α production through the P38 and JNK pathways^53,54^. In chronic alcohol consumption, as observed in alcoholic patients and mouse models, the production of adiponectin is decreased, but leptin production is increased, resulting in damage to hepatocytes by high concentrations of TNF-α^55^. Furthermore, adipose tissue influences alcoholic liver injury by modulating the cargo of the secreted EVs trafficking to the liver via the secretome^56^.

## The gut–liver axis in ALD

### Gut microbiota and the immune system

In addition to energy absorption, the gut has a well-established immune system that plays a role in homeostasis. Mechanochemically, the surface of enterocytes in the small intestine consists of microvilli coated with a glycocalyx of mucin, which produces diverse enzymes for the defense against antigens or pathogens from the lumen^57^. The intestinal lamina propria consists of various types of immune cells and lymphatic tissues. For example, gut-associated lymphoid tissue and mesenteric lymph nodes, including Peyer’s patches, are composed of numerous T cells and B cells, and naive lymphocytes are primed by pathogens or antigen-presenting cells that are external to the immune response^58^. Several subsets of dendritic cells regulate T-cell stimulation and suppression by recognizing pathogens through TLR signaling; in addition, macrophages are involved in the phagocytosis of dead cells and induce proliferation of regulatory T cells by producing IL-10^57,59^. Interestingly, the gut microbiome coexists with its host; there are over 100 trillion microorganisms in the human gastrointestinal tract, and the microbiome not only comprises bacteria, but also fungi, archaea, protists, and viruses^60^. The microbiome is established by the influence of environmental conditions and food consumption after birth and is important for immune and metabolic homeostasis of the host. However, the composition of the microbiome changes in ALD^61^.

### Metabolic effects of alcohol on gut microbiota and immunity

Ingested alcohol is absorbed and diffused in the gastrointestinal tract, where the stomach and the proximal small intestine are responsible for ~20% and 70% of its absorption, respectively^62^. Among alcohol dehydrogenases (ADHs), ADH1A is expressed in the small intestine and enables alcohol metabolism, whereas the other isoenzymes have a role in vitamin A (retinol) metabolism, which is essential for intestinal epithelial proliferation or differentiation^63^. However, high concentrations of alcohol and chronic absorption are metabolized by CYP2E1, and oxidative stress and byproducts caused by alcohol metabolism alter the intestinal tight junction proteins (e.g., occludin and zonula occludens-1) and adherent junction proteins (e.g., β-catenin and E-cadherin), which interconnect the epithelial cells that have a role in intestinal barrier integrity^63–65^. In addition, CYP2E1-mediated alcohol metabolism increases intestinal permeability by inducing the expression of circadian clock proteins such as circadian locomotor output cycles kaput and period circadian clock 2^66^. Furthermore, chronic alcohol consumption destroys the intestinal epithelial barrier, leading to changes, such as overgrowth and dysbiosis, in the intestinal microbiome of rodents and patients. Alcohol-mediated dysbiosis increases the levels of unconjugated bile acids in the gut, which reduces farnesoid X receptor activity and fibroblast growth factor (FGF)-15 expression in enterocytes, leading to increased bile acid concentration in the blood through upregulated CYP7A1 expression in hepatocytes^67^. However, treatment with the FXR agonist, fexaramine, or overexpression of FGF-15 leads to recovery of the gut barrier and attenuates ALD^67^.

Overgrowth of gram-negative bacteria owing to alcohol consumption induces endotoxin production and release in the blood of rodents and human patients^68^. In addition, LPS enhances intestinal permeability by producing nitric oxide by autocrine signaling, thereby activating myosin light chain kinase and the expression of TLR4 and CD14 in enterocytes^69,70^. In alcohol-fed mice, bacteria of the phyla *Verrucomicrobia* and *Bacteroidetes* increase, whereas those of the phylum *Firmicutes* decrease^71^. In alcoholic patients, *Proteobacteria* and *Firmicute* phyla increase; however, this increase depends on the stage of liver disease^61,72^. A recent study reported that the numbers of cytolysin-positive *Enterococcus faecalis* correlate with the severity of alcoholic hepatitis and mortality^73^. Interestingly, intestinal overgrowth of *K. pneumoniae* causes fatty liver disease because these bacteria produce alcohol, in the absence of alcohol consumption^74^. Moreover, populations of fungi, as well as bacteria, are increased in the gut owing to alcohol consumption and translocate to the liver to induce inflammation through the β-glucan-CLEC7A axis^75^.

### Inflammation in the gut

Increasing intestinal permeability leads to translocation of bacteria and PAMPs to the portal blood and exposure to the intestinal immune system, wherein they stimulate myeloid cells and induce systemic inflammation^76^. However, in ALD, the exact underlying mechanisms of gut inflammation by alcohol are still unclear. In mice with chronic exposure to alcohol, the expression of pro-inflammatory mediators, such as TNF-α, IL-1β, IL-6, and iNOS, increases in the distal ileum, and the anti-inflammatory cytokine IL-11 also increases significantly^77^. In an “alcohol combined with burn injury” model, intestinal macrophages or monocytes increase the production of TNF-α, prostaglandin E_2_, and IL-10 and decrease the expression of MHC class II and antigen presentation^78^. In addition, in this model, increased IL-18 has a critical role in the recruitment and activation of neutrophils in the damaged intestines of rats^79^. Regarding adaptive immune responses, acute alcohol administration to mice depletes T cells and B cells in the MLN^80,81^. IL-12, which has key roles in Th1 differentiation and IFN-γ production, is reduced in rat T cells after alcohol intoxication and burn injury^82^.

### Cross-talk between the liver and gut

In ALD, gut-liver cross-talk is mainly caused by increased gut permeability, leading to the entry of PAMPs (e.g., LPS) into the liver through the portal circulation. LPS binds to TLR4 in combination with CD14, MD-2, and LPS-binding protein, and its signal is delivered by the recruitment of adapter molecules, such as MyD88 and TRIF, in Kupffer cells and macrophages^83^. MyD88-mediated NF-kB activation produces pro-inflammatory cytokines (e.g., TNF-α, IL-6, and IL-1β) and the chemokine CCL2, whereas the TRIF pathway induces the production of type-I interferons^84^. Thus, both TLR4 and CD14 are considered therapeutic targets for ALD. Moreover, TLR4 is expressed not only in immune cells but also in hepatocytes and HSCs. In hepatocytes, TLR4-LPS activates the NF-kB pathway and pro-inflammatory signaling, leading to increased expression of SREBP1c that further results in steatosis or hepatic injury^55^. In response to LPS, HSCs release pro-inflammatory cytokines (e.g., TNF-α, IL-6, and IL-8), chemokines (e.g., CCL2, ICAM-1, RANTES, and CCR5) and adhesion molecules^84^. Furthermore, TNF-α production in Kupffer cells and the recruitment of immune cells by LPS/TLR4 activate HSCs and induce liver fibrosis by producing TGF-β and extracellular matrix^85^.

## The BM–liver axis in ALD

### Cross-talk between the liver and BM

In addition to tissue-resident immune cells, such as Kupffer cells, most inflammatory cells are derived from the BM in alcoholic inflammation of adipose tissue, the gut, and the liver. The BM is thought to be an immunoregulatory organ that has a role not only in hematopoiesis but also in immune responses^86^. Thus, inflammatory cells mature and proliferate in the BM and egress into the bloodstream by the gradients of cytokines and chemokines, such as CXCL12-CXCR4, CXCL1-CXCR2, and CCL2-CCR2^87,88^. Clinical and experimental studies have shown that autologous or allogenic BM cell transplantation is effective for liver cirrhosis and fibrosis in patients and mice, respectively, in which migrated BM cells improve impaired liver functions in patients or stimulate IL-10 production in Gr1^+^CD11b^+^ cells in mice^89,90^. However, autologous BM cell transplantation did not show beneficial effects in liver function or IL-10 production in a patient with alcoholic cirrhosis^90^, suggesting that chronic alcohol consumption might affect BM cells. Recently, granulocyte colony-stimulating factor (G-CSF), a glycoprotein that differentiates and matures stem cells into granulocytes in the BM, has emerged as a treatment candidate for alcoholic hepatitis in several countries^91–94^. Clinical and experimental studies have shown that G-CSF treatment improves alcoholic hepatitis by migrating CD34^+^ hematopoietic stem cells and stimulating hepatocyte regeneration in patients and mice, suggesting a novel therapeutic effect of G-CSF on alcoholic hepatitis^91,95^.

In contrast, the effects of alcohol intake on the BM have yet to be clearly elucidated. Intriguingly, our recent study suggests that BM-derived monocytes can be differentiated into F4/80^high^ Kupffer-like cells in a CX_3_CR1-dependent manner and have pro-inflammatory functions in ALD^96^. Similarly, other studies have demonstrated that monocytes are recruited by liver HSCs and liver sinusoidal endothelial cells, and they differentiate into monocyte-derived Kupffer cells through the NOTCH-BMP pathway in certain situations^97,98^. These studies suggest that BM-derived macrophages may have a role in ALD through the differentiation of Kupffer-like cells. Although the exact functions of such macrophages are not yet clear, they may have various functions depending on multiple subtypes of macrophages in the BM and various conditions in ALD. Given that alcohol metabolism occurs within the BM, clarification of alcohol-metabolizing cells and their roles in ALD are likely to be of vital importance in the treatment of ALD. Consequently, further studies on this subject are of the utmost importance.

## Future perspectives

Although the best way to prevent further progression of ALD is to abstain from consuming alcohol, current therapies for ALD focus on the stages of disease severity and mostly depend on steroid treatments. However, we are still struggling to use such treatment strategies in ALD patients who consume high amounts of alcohol and are resistant to medication. Thus, there is an urgent need to develop alternative approaches to treat ALD. Generally, alcohol metabolism is considered to occur in the liver, but recent studies have demonstrated that other organs, including adipose tissue and intestine, can metabolize alcohol partially owing to the expression of ADHs and CYP2E1. In addition, mechanical pathways related to oxidative stress-mediated inflammation and injury are well known to occur in adipose tissue and the gut. Furthermore, organ cross-talk is triggered by the entry of inflammatory cytokines and molecules, such as adipokines, DAMPs, and PAMPs, as well as the migration of pro-inflammatory cells into the liver, which further exacerbates ALD by activating hepatic immune cells and inducing hepatocyte injury. As a result, the entire paradigm of ALD originates not only due to liver injury but also due to cross-talk with other organs. Therefore, using a single factor in a specific organ as a therapeutic target may be ineffective, and combination therapy aimed at multiple organs is likely required for the treatment of ALD.
